# Supplementary material for: In Situ Construction of a MgSn(OH)6 Perovskite/SnO2 Type-II Heterojunction: A Highly Efficient Photocatalyst towards Photodegradation of Tetracycline
Source: Nanomaterials (Basel). 2019 Dec 24;10(1):53. doi: 10.3390/nano10010053 (PMC7023148; doi:10.3390/nano10010053)
Supplement: Supplementary file 1 [file nanomaterials-10-00053-s001.pdf]

nanomaterials

MDPI

Article

In Situ Construction of a $MgSn(OH)_6$ Perovskite/SnO_2 Type-II Heterojunction: A Highly Efficient Photocatalyst towards Photodegradation of Tetracycline

Yuanyuan Li [1,*], Xiaofang Tian [1], Yaoqiong Wang [2], Qimei Yang [1], Yue Diao [1], Bin Zhang [3,*], and Dingfeng Yang [2,*]

1 Department of Biological and Chemical Engineering, Cooperative Innovation Center of Lipid Resources and Children's Daily Chemicals, Chongqing University of Education, Chongqing 400067, China; tianxiaofang0717@163.com (X.T.); yang53075@163.com (Q.Y.); yuediao704@163.com (Y.D.)
2 College of Chemistry and Chemical Engineering, Chongqing University of Technology, 69 Hongguang Rd., Lijiatuo, Banan District, Chongqing 400054, China; wangyq863@cqut.edu.cn (Y.W.)
3 Analytical and Testing Center of Chongqing University, Chongqing 401331, China; welon5337@126.com
* Correspondence: liyy@cque.edu.cn (Y.L.); welon5337@126.com (B.Z.); yangxunscience@cqut.edu.cn (D.Y.); Tel.: +86-1590-230-5519 (Y.L.); +86-1992-370-1911 (D.Y.); +86-1520-158-5493 (B.Z.)

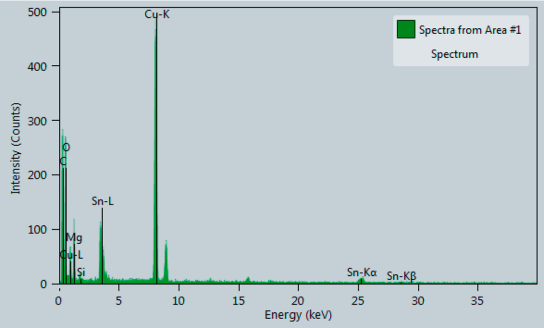

Figure S1. elemental mapping of heterojunction MSOH-SO-3.

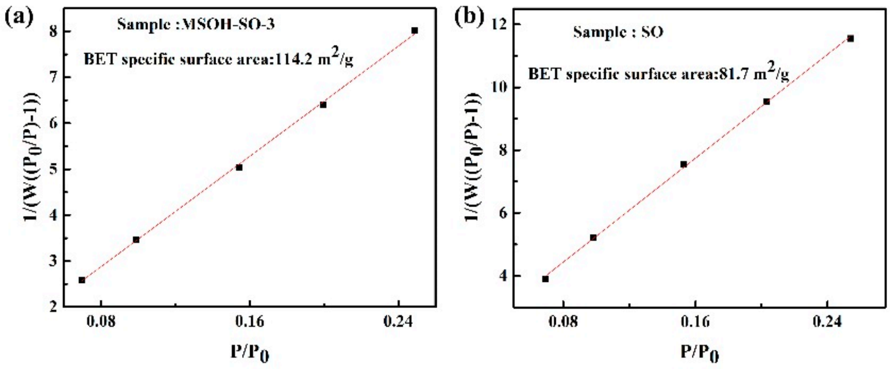

Figure S2. The specific surface area determination performed by BET measurement with N_2 adsorption-desorption. (a) Heterojunction MSOH-SO-3 (b) SnO_2 (SO).

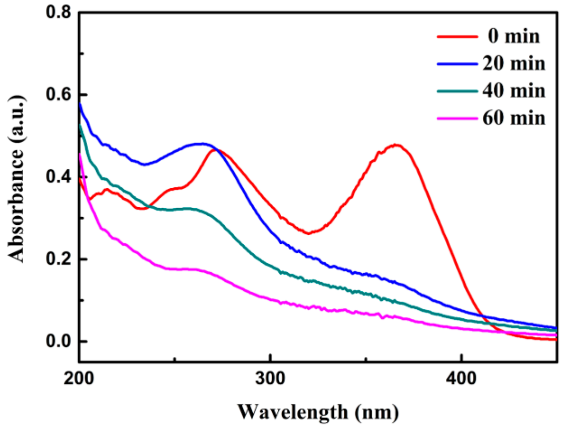

Figure S3. UV-visible absorption of TC as a function of irradiation time over the heterojunction MSOH-SO-3.

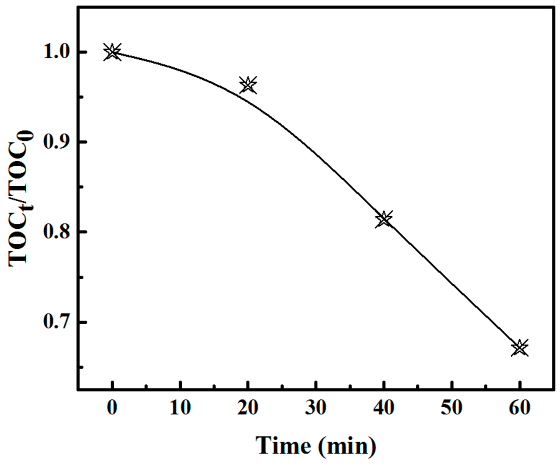

Figure S4. The TOC degradation of TC solution under different UV-light irradiation.(TOC_0: initial TOC value; TOC_t: TOC value at photodegradation time t).

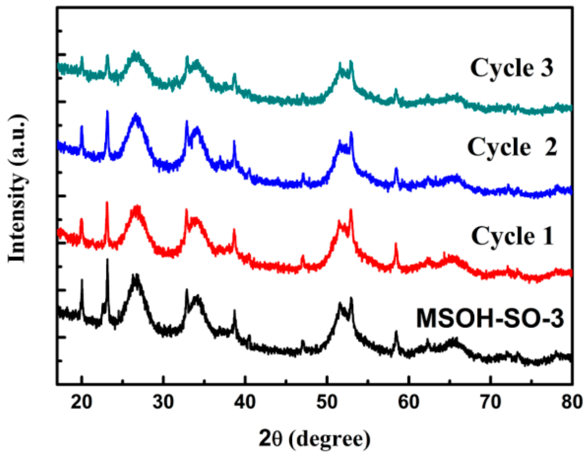

Figure S5. XRD patterns of sample MSOH-SO-3 of cycling runs on the photocatalytic reaction of degrading TC solution.

© 2019 by the authors. Submitted for possible open access publication under the terms and conditions of the Creative Commons Attribution (CC BY) license (http://creativecommons.org/licenses/by/4.0/).
